# *S*-equol Exerts Estradiol-Like Anorectic Action with Minimal Stimulation of Estrogen Receptor-α in Ovariectomized Rats

**DOI:** 10.3389/fendo.2017.00281

**Published:** 2017-10-19

**Authors:** Yuri Nishimura, Kaori Mabuchi, Azusa Takano, Yayoi Hara, Hiroko Negishi, Keiko Morimoto, Tomomi Ueno, Shigeto Uchiyama, Akira Takamata

**Affiliations:** ^1^Department of Environmental Health, Nara Women’s University, Nara, Japan; ^2^Saga Nutraceuticals Research Institute, Otsuka Pharmaceutical Co., Ltd., Saga, Japan

**Keywords:** *S*-equol, estradiol, hypophagic effect, circadian feeding rhythm, suprachiasmatic nucleus, estrogen receptor-α

## Abstract

Chronic estrogen replacement in ovariectomized rats attenuates food intake and enhances c-Fos expression in the suprachiasmatic nucleus (SCN), specifically during the light phase. *S*-equol, a metabolite of daidzein, has a strong affinity for estrogen receptor (ER)-β and exerts estrogenic activity. The purpose of the present study was to elucidate whether *S*-equol exerts an estrogen-like anorectic effect by modifying the regulation of the circadian feeding rhythm in ovariectomized rats. Ovariectomized female Wistar rats were divided into an estradiol (E2)-replaced group and cholesterol (vehicle; Veh)-treated group. These animals were fed either a standard diet or an *S*-equol-containing diet for 13 days. Then, the brain, uterus, and pituitary gland were collected along with blood samples. In the rats fed the standard diet, E2 replacement attenuated food intake (*P* < 0.001) and enhanced c-Fos expression in the SCN (*P* < 0.01) during the light phase. Dietary *S*-equol supplementation reduced food intake (*P* < 0.01) and increased c-Fos expression in the SCN (*P* < 0.01) in the Veh-treated rats but not in the E2-replaced rats during the light phase. Dietary *S*-equol did not alter ER-α expression in the medial preoptic area or the arcuate nucleus, nor did dietary S-equol affect pituitary gland weight or endometrial epithelial layer thickness. By contrast, E2 replacement not only markedly decreased ER-α expression in these brain areas (*P* < 0.001) but also increased both the pituitary gland weight (*P* < 0.001) and the endometrial epithelial layer thickness (*P* < 0.001). Thus, dietary *S*-equol acts as an anorectic by modifying the diurnal feeding pattern in a manner similar to E2 in ovariectomized rats; however, the mechanism of action is not likely to be mediated by ER-α. The data suggest a possibility that dietary *S*-equol could be an alternative to hormone replacement therapy for the prevention of hyperphagia and obesity with a lower risk of adverse effects induced by ER-α stimulation.

## Introduction

The prevalence of obesity increases after menopause, which can elevate the risk of metabolic and cardiovascular diseases (1–5). Estrogen but not progesterone has been shown to exert antiobesity and anorectic effects (1, 6, 7). The anorectic action of estrogen is mediated by central estrogen receptors (ERs) (8). Recent studies have demonstrated that ER-α is mainly involved in estrogen-induced hypophagia (8–11), although ER-β has also been reported to be involved in estrogen-induced hypophagia (12).

Hormone replacement therapy (HRT) is widely prescribed to menopausal women for the relief of perimenopausal symptoms, and it has been shown to be effective for the prevention of obesity, hyperphagia, and metabolic syndrome in postmenopausal women (5, 13, 14). However, HRT has adverse effects, including an elevated risk of venous thromboembolism and breast, endometrial, and ovarian cancers (2, 15). As an alternative to HRT, a diet rich in soy has been shown to have beneficial effects on the health of menopausal women (16) because soy isoflavones reportedly act as ER modulators (17, 18).

*S*-equol is a metabolite of daidzein produced by enterobacteria that are present in approximately 25–50% of individuals (19–21). Reportedly, *S*-equol exerts relatively strong estrogenic effects and has a higher affinity for ER-β (20). Studies of postmenopausal women and ovariectomized rats have shown that *S*-equol administration reduces hot flashes (22), osteoporosis (23, 24), body weight (22, 25, 26) and depressive behavior (25) and increases glucose tolerance (26, 27). However, little is known about the effect of *S*-equol on feeding behavior. Several studies have reported that *S*-equol reduces food intake (22, 25), but these studies did not examine the anorectic effect in detail; additionally, the mechanism of action of *S*-equol on feeding behavior has not yet been clarified.

We previously reported that estradiol (E2) replacement in ovariectomized rats attenuates food intake and enhances c-Fos expression in the suprachiasmatic nucleus (SCN), the center for circadian rhythm regulation, specifically during the light phase (28). These data suggest that modification of circadian feeding rhythm regulation is one of the main factors underlying estrogen-induced hypophagia (28, 29). Based on these observations and on the anorectic effect of *S*-equol, we hypothesized that *S*-equol would also attenuate food intake and enhance c-Fos expression in the SCN, specifically during the light phase.

E2 replacement in ovariectomized rats reportedly downregulates ER-α expression in brain regions, including the medial preoptic area (MPO) and arcuate nucleus (Arc) (30). However, the effect of *S*-equol on ER-α expression in the brain remains unknown. In the present study, we hypothesized that dietary *S*-equol would not induce ER-α downregulation in the brain. We further hypothesized that *S*-equol would exert no hypertrophic actions on the uterus or pituitary gland, where ER-α stimulation causes hypertrophy (30–32), because *S*-equol has a higher affinity for ER-β (21).

To elucidate whether dietary *S*-equol reduces the food intake of ovariectomized rats similar to E2 replacement by adjusting the circadian feeding rhythm, we examined the effects of dietary *S*-equol on the body weight, circadian feeding rhythm and c-Fos expression rhythm in the SCN of ovariectomized rats with and without E2 replacement. Furthermore, to elucidate the involvement of ER-α on the actions of *S*-equol, we examined the effect of *S*-equol on the expression of ER-α in the MPO and Arc, the pituitary gland weight, and the thickness of the endometrial epithelial layer.

## Materials and Methods

All experiments and animal care procedures were conducted in accordance with the guidelines for animal care and use of Nara Women’s University. All experimental procedures were approved by the Animal Care and Use Committee of Nara Women’s University.

### Experimental Design

To elucidate the effects of E2 replacement and dietary *S*-equol, seven-week-old ovariectomized Wistar rats (Jcl:Wistar, CLEA, Japan) were assigned to one of four experimental groups (2 × 2 design): E2-replaced rats fed a standard diet (ConD) (E2 + ConD; *n* = 10); E2-replaced rats fed an *S*-equol-containing diet (EqD) (E2 + EqD; *n* = 10); vehicle (Veh)-treated rats fed ConD (Veh + ConD; *n* = 11); and Veh-treated rats fed EqD (Veh + EqD; *n* = 12).

### Surgery

All rats were bilaterally ovariectomized from a dorsal approach under general anesthesia with pentobarbital sodium (50 mg/kg, i.p.; Somnopentyl; Kyoritsu Pharmaceutical, Tokyo, Japan) and sevoflurane (2%; Sevofran; Maruishi Pharmaceutical, Osaka, Japan). After the ovariectomy, a silicon capsule filled with a mixture of 17β-E2 (Sigma-Aldrich Japan, Tokyo, Japan) and cholesterol (Sigma-Aldrich Japan, Tokyo, Japan) powders (E2:cholesterol = 1:4 by weight) was implanted subcutaneously in E2-replaced rats, and a capsule containing cholesterol alone was implanted in vehicle-treated rats through an interscapular incision. The capsule was made from silicon tubing (ID, 2 mm; OD, 3 mm; inner length, 20 mm; ARAM Co., Osaka, Japan), and both ends of each capsule were sealed with silicon polymer bulking agents (Bath Caulk N; Cemedine, Tokyo, Japan). Our preliminary experiment demonstrated that this E2 replacement procedure elevates the plasma E2 concentration to the level that is comparable to the level during proestrus, and induces a relatively strong hypophagic effect. Following surgery, all rats were housed individually in a plastic cage in a chamber with a controlled ambient temperature of 23°C and a relative humidity of 40% under a 12/12 h light/dark cycle in which the lights were switched on at 0700 h. The illumination intensity was ~250 lx on the bottom of the cages.

### Diet

After the surgery, the rats were fed either a standard (control) diet or an EqD. The standard diet was a modified pellet-formed AIN-93M diet in which soybean oil was replaced with corn oil (Oriental Yeast, Osaka, Japan). The EqD was a diet in which *S*-equol-containing SE5-OH (Otsuka Pharmaceutical, Tokyo, Japan) was added to the standard diet. The protein and lipid contents of the EqD were adjusted to values equivalent to those of the standard diet (Table 1). SE5-OH was developed by the fermentation of soy germ by the *S-*equol-producing lactic acid bacterium *Lactococcus* strain 20-92. The major characteristics of SE5-OH are the relatively high content of *S*-equol (0.5–0.6%) as an isoflavonoid (total isoflavonoid content is approximately less than 1%) and the low genistein content (33). The EqD contained 138.7 mg/kg *S*-equol and 14.0 mg/kg genistein. The contents of isoflavones and metabolites in SE5-OH and EqD are shown in Table S1 in Supplementary Material.

**Table 1:** Compositions of the standard (control) diet (ConD) and the *S*-equol-containing diet (EqD).

	ConD (g/kg diet)	EqD (g/kg diet)
**Nutritional composition**		
Protein	141.8	141.8
Carbohydrate	721	714
Fat	40	40
Fiber	50	50
**Ingredient**		
Casein	140	131
l-Cystine	1.8	1.8
Cornstarch	466	457
Pregelatinized cornstarch	155	155
Sucrose	100	100
Corn oil	40	37
Cellulose powder	50	48
AIN-93M mineral mixture	35	35
AIN-93 vitamin mixture	10	10
Choline bitartrate	2.5	2.5
*Tert*-butylhydroquinone	0.008	0.008
SE5-OH	–	23

*Prepared according to the AIN-93M formulation*.

*SE5-OH, a fermented soy germ product containing S-equol*.

### Protocol

Starting the day after the ovariectomy and E2 replacement/Veh treatment, the rats were fed either the control or EqD for 13 days. For six of the rats in each group, continuous food intake and locomotor activity measurements were initiated after a 6-day postoperative recovery period, and the measurements were conducted for 7 days. The body weight of all rats was measured manually at 0900 h using an electric balance.

On the 13th day after the ovariectomy, the rats were deeply anesthetized with 2% sevoflurane and pentobarbital (100 mg/kg; i.p.) at 0900 h [Zeitgeber time 2 h (ZT2)] (*n* = 5 in the E2 + ConD and E2 + EqD groups, *n* = 6 in the Veh + ConD group, and *n* = 7 in the Veh + EqD group) or at 2100 h (ZT14) (*n* = 5 in each group), and blood samples were collected by cardiac puncture. The sampled blood was transferred into chilled EDTA-containing tubes and centrifuged immediately; the separated plasma was stored at −60°C until assays for plasma E2 and glucose were performed. After the blood sampling, each rat was transcardially perfused with ice-cold phosphate-buffered saline (PBS; pH 7.4) for exsanguination and then with 4% paraformaldehyde in phosphate buffer (0.1 M; pH 7.4) for fixation; subsequently, the brain was removed. All experimental procedures starting at ZT14 were performed under dim red light. After removing the brain, the pituitary gland was removed from the hypophyseal fossa, and the wet weight of the pituitary gland was measured using an electric balance. Then, the uterus was excised and immersed in 4% paraformaldehyde in phosphate buffer for fixation.

### Behavioral Measurements and Plasma E2 and Glucose Assays

The food intake measurements for six of the rats in each group were performed using an automated feeding monitoring apparatus (FIS-001 and FIC-001; Muromachi, Tokyo, Japan). The locomotor activity of the same rats was measured using an infrared activity monitor (Kyoto L Giken, Kyoto, Japan). However, this apparatus only measured horizontal movement.

The plasma E2 concentration was measured in duplicate using an E2 EIA Kit (Cayman Chemical Company, Ann Arbor, MI, USA). Plasma glucose was measured in duplicate by an enzymatic method (Glucose CII-test Wako; Wako Pure Chemical, Osaka, Japan).

### Histological Procedures

The removed brains were postfixed in fixative at 4°C and then immersed in PBS containing 15% sucrose for 1 day, followed by 25% sucrose for 2 days for cryoprotection. Then, the brain was coronally sliced into 30-μm thick sections using a cryostat (Leica CM3050 S; Leica Biosystems, Wetzlar, Germany).

Immunohistochemical staining for c-Fos in the SCN was performed on free-floating sections as previously reported (28), and ER-α staining in the MPO and Arc was performed using the same protocol as c-Fos staining; however, the primary antibody against ER-α was used. Briefly, after incubation with rabbit c-Fos antibody (1:4,000; Sc52; Santa Cruz Biotechnology, Dallas, TX, USA) for the SCN or with rabbit ER-α antibody (1:1,000; Sc7207; Santa Cruz Biotechnology, Dallas, TX, USA) for the MPO and Arc, the sections were incubated in biotinylated goat antirabbit IgG (dilution 1:400; BA-100; Vector Laboratories, Burlingame, CA, USA), followed by ABC Elite Kit solution (dilution 1:400; Vector Laboratories, Burlingame, CA, USA). Visualization of the antibodies was performed using 0.02% 3,3-diaminobenzidine (Dojindo Laboratories, Kumamoto, Japan) and 0.01% H_2_O_2_ in 50 mM Tris HCl buffer (pH 7.4). The sections were mounted on gelatin-coated glass slides, dehydrated with graded ethanol, cleared with Lemosol^®^, and coverslipped.

After cryoprotection, part of the fixed uterus 10–15 mm from the root of the horn was excised and cross-sectionally sliced into 20-μm thick sections using a cryostat. The sample was then mounted on amino silane (APS)-coated glass slides (Matsunami Glass, Osaka, Japan). The mounted sections were stained with hematoxylin and eosin, dehydrated with graded ethanol, cleared with Lemosol^®^, and coverslipped.

### Data Analyses

ER-α expression in the unilateral MPO (two sections) and Arc (two sections) and c-Fos expression in the unilateral SCN (three sections) were examined using a microscope (Olympus BX-51; Olympus Corp., Tokyo, Japan). Images of these areas were obtained using a cooled CCD camera (Retiga 4000-R; QImaging, Canada). The MPO, Arc, and SCN were identified using the rat brain stereotaxic atlas, and sections were carefully matched across all animals in all experimental groups (34).

The numbers of ER-α-immunoreactive (ir) cells in an area (315 × 315 µm) of the MPO and in the whole Arc were counted in each section using image analysis software (ImageJ; NIH, Bethesda, MD). The number of c-Fos immunoreactive (c-Fos-ir) nuclei in the SCN was counted in the whole SCN and in the ventrolateral region of the SCN in three sections. Further analysis was performed using the mean numbers of ER-α-ir and c-Fos-ir cells in the sections from each rat.

### Statistical Analysis

The data are shown as the mean ± standard error of the mean (SEM). The values obtained for food intake and locomotor activity in each animal are presented as the mean of measurements taken over 7 days after a 6-day recovery period. The plasma E2 and glucose concentrations, ER-α-ir nuclei count, pituitary gland weight, and endometrial epithelial layer thickness are shown as the mean ± SEM of these values obtained from all rats in each group, i.e., including data from samples obtained during both the light and dark phases, because these values did not differ between the light and dark phases in any group.

Three-way analysis of variance (ANOVA) with repeated measures (two between factor and one within factor) was used to determine the effect of E2-repacement, dietary *S*-equol and time of body weight change. Two-way ANOVA with two between factors was used to determine the effects of E2 replacement and dietary *S*-equol. Levene’s test was performed to test the homogeneity of variances before ANOVA. Log-transformation was performed for plasma E2 concentration, pituitary gland weight, and endometrial epithelial layer thickness before performing ANOVA because a relatively larger effect, and therefore a larger variance, of E2 replacement resulted in a difference in the variance of these variables among the groups. Differences between data of specific interest were determined using the Tukey HSD *post hoc* test. *P* < 0.05 was considered statistically significant.

## Results

### E2 Concentration

The plasma E2 concentration was higher in the E2-replaced rats than in the Veh-treated rats (*P* < 0.001). The plasma E2 concentration did not differ between the E2 + ConD (73.6 ± 10.3 pg/ml) and E2 + EqD (90.9 ± 28.6 pg/ml) groups, or between the Veh + ConD (38.6 ± 6 pg/ml) and Veh + EqD (28.6 ± 4.1 pg/ml) groups. The plasma E2 concentration was within the physiological range for rats, which is comparable to the level during the proestrus (35).

### Body Weight

**Figure 1:**
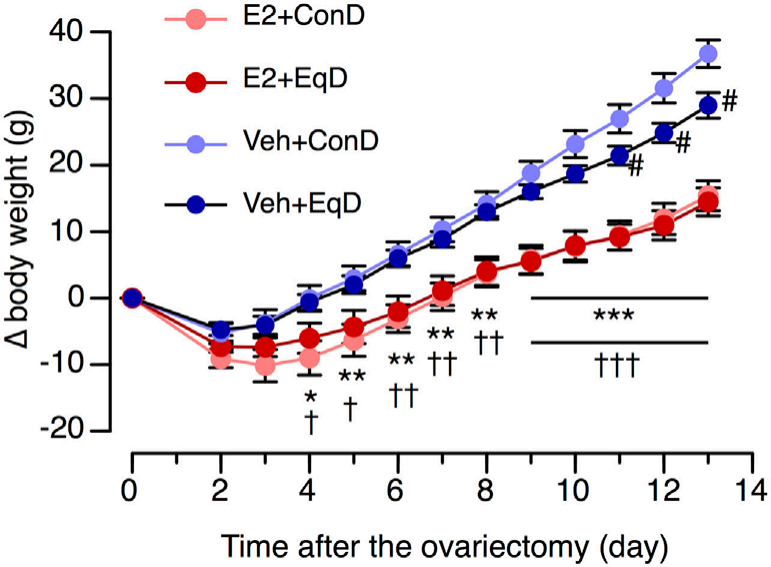
The effect of estradiol (E2) replacement and dietary *S*-equol on the time course of body weight change (Δ body weight) from the day of ovariectomy in ovariectomized rats. Rats were ovariectomized and assigned to the E2-replaced or cholesterol [vehicle (Veh)]-treated group. These rats were fed a standard (control) diet (ConD) or an *S*-equol-containing diet (EqD). Values are shown as the mean ± SEM (*n* = 10 in E2 + ConD; *n* = 10 in E2 + EqD; *n* = 11 in Veh + ConD; *n* = 12 in Veh-EqD). *, **, and ***, significant differences (*P* < 0.05, *P* < 0.01, and *P* < 0.001, respectively) between the E2 + ConD and Veh + ConD groups. ^†^, ^††^, and ^†††^, significant differences (*P* < 0.05, *P* < 0.01, and *P* < 0.001, respectively) between the E2 + EqD and Veh + EqD groups. ^#^, significant differences (*P* < 0.05) between the Veh + ConD and Veh + EqD groups.

The body weight before ovariectomy did not differ among the groups: 143 ± 3.3 g in the E2 + ConD group, 144.5 ± 3.3 g in the E2 + EqD group, 142.3 ± 2.9 g in the Veh + ConD group, and 145.5 ± 3 g in the Veh + EqD group. The increase in body weight over the experimental period was much larger in the Veh-treated rats than in the E2-replaced rats (Figure 1). The increase in body weight on the 13th day after ovariectomy was significantly greater in the Veh + ConD group than in the E2 + ConD group (*P* < 0.001). The increase was significantly lower in the Veh + EqD group than in the Veh + ConD group (*P* < 0.01), whereas the increase did not differ between the E2 + EqD and E2 + ConD groups (Figure 1).

### Food Intake and Locomotor Activity

**Figure 2:**
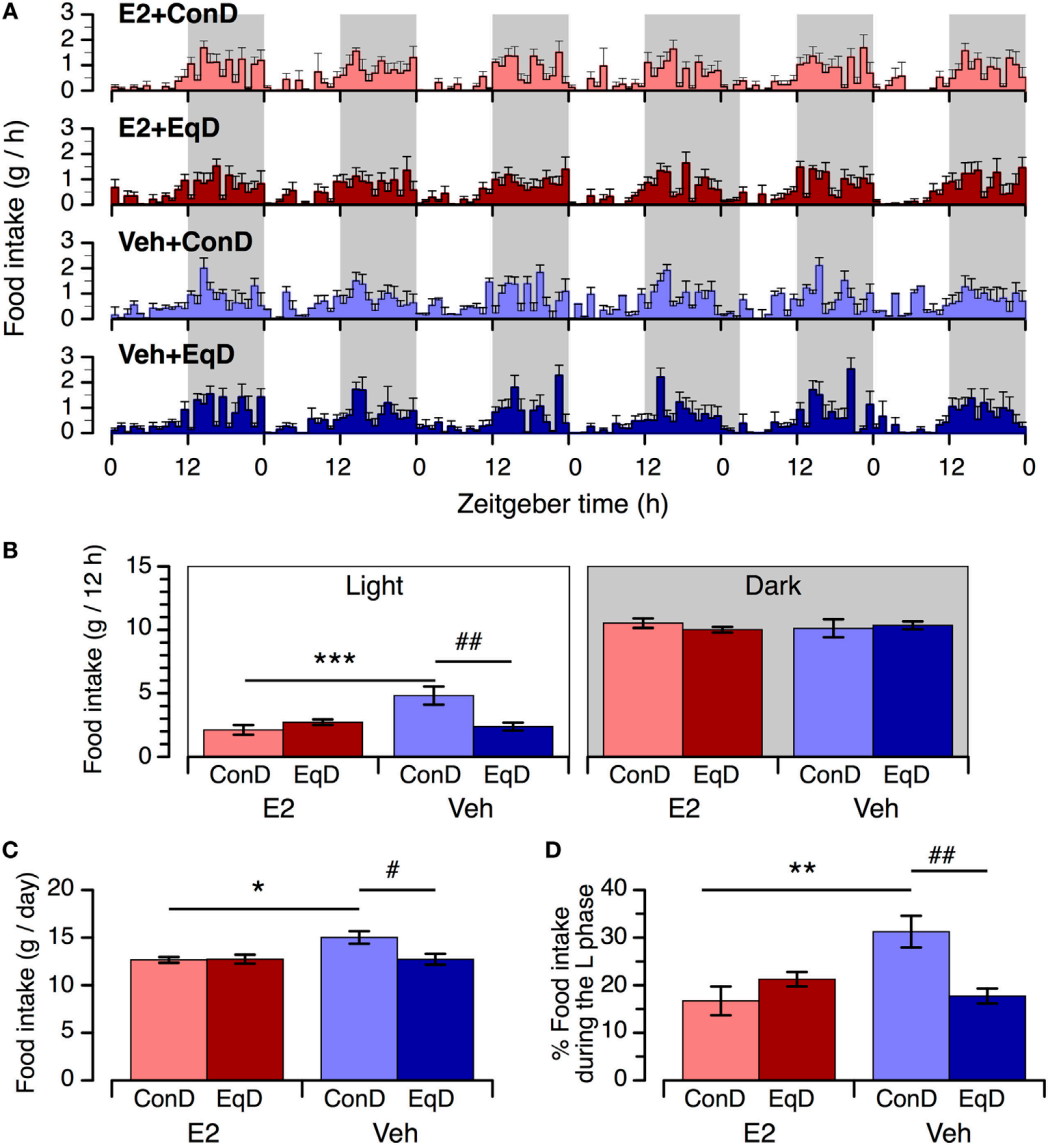
The effect of estradiol (E2) replacement and dietary *S*-equol on circadian feeding patterns **(A)**, food intake during the light phase [**(B)**, left] and dark phase [**(B)**, right], daily food intake **(C)**, and percentage of daily food intake that occurred during the light phase **(D)** in ovariectomized rats. Rats were ovariectomized and assigned to the E2-replaced or cholesterol [vehicle (Veh)]-treated group. These rats were fed a standard (control) diet (ConD) or an *S*-equol-containing diet (EqD) Values are shown as the mean ± SEM (*n* = 6 in each group). Shading in A depicts the 12-h dark phase. *, **, and ***, significant differences (*P* < 0.05, *P* < 0.01, and *P* < 0.001, respectively) between the E2-replaced and Veh-treated groups fed the same diet. ^#^ and ^##^, significant differences (*P* < 0.05 and *P* < 0.01, respectively) between the rats fed ConD and EqD with the same treatment.

The animals in all groups showed a clear circadian feeding rhythm, with lower intake during the light phase and higher intake during the dark phase (Figure 2A). However, the circadian feeding rhythm was relatively disturbed in the Veh + ConD group compared with the other groups (Figure 2A).

During the light phase, food intake was significantly greater in the Veh + ConD group than in the E2 + ConD group (*P* < 0.001) (Figure 2B, left). Food intake during the light phase was significantly lower in the Veh + EqD group than in the Veh + ConD group (*P* < 0.01), whereas food intake in the E2 + EqD group did not differ from that in the E2 + ConD group, and food intake in the Veh + EqD group was similar to that in the E2 + ConD and E2 + EqD groups (Figure 2B, left). During the dark phase, food intake did not differ among the four groups (Figure 2B, right).

Daily food intake and the percentage of daily food intake during the light phase were significantly greater in the Veh + ConD group than in the E2 + ConD group (*P* < 0.05 and *P* < 0.01, respectively) (Figures 2C,D). Daily food intake and the percentage of daily food intake during the light phase were lower in the Veh + EqD group than in the Veh + ConD group (*P* < 0.05 and *P* < 0.01, respectively), whereas these variables did not differ between the E2 + EqD and E2 + ConD groups and were similar between the E2 + EqD and Veh + EqD groups (Figures 2C,D).

**Figure 3:**
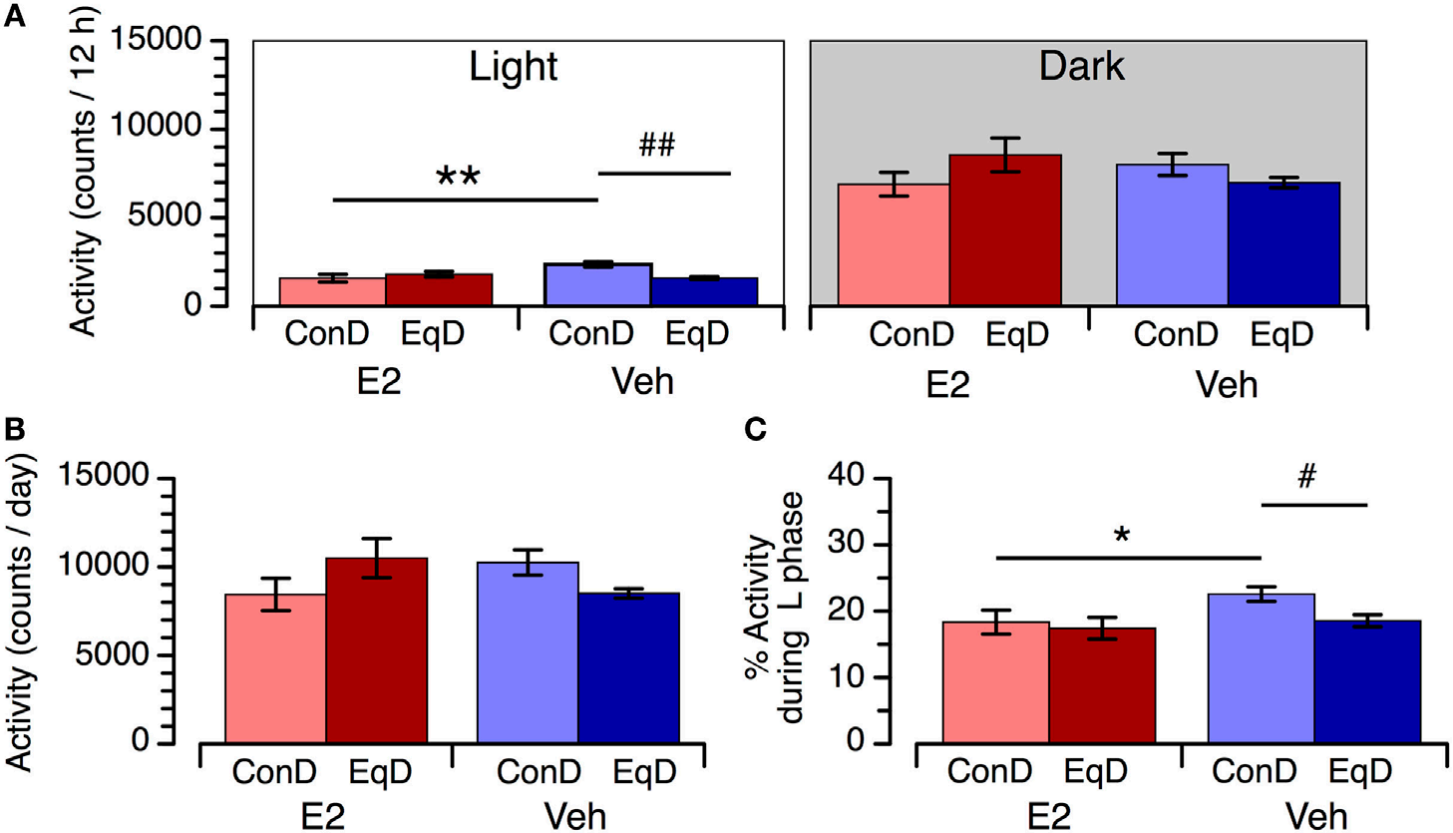
The effect of estradiol (E2) replacement and dietary *S*-equol on locomotor activity during the light phase [**(A)**, left] and dark phase [**(A)**, right], daily activity **(B)**, and percentage of daily activity during the light phase **(C)** in ovariectomized rats. Rats were ovariectomized and assigned to E2-replaced and cholesterol [vehicle (Veh)]-treated groups. These rats were fed a standard (control) diet (ConD) or an *S*-equol-containing diet (EqD). Values are shown as the mean ± SEM (*n* = 6 in each group). * and **, significant differences (*P* < 0.05 and *P* < 0.01, respectively) between the E2-replaced and Veh-treated rats fed the same diet; ^#^ and ^##^, significant differences (*P* < 0.05 and *P* < 0.01, respectively) between the rats fed ConD and EqD with the same treatment.

During the light phase, locomotor activity was significantly greater in the Veh + ConD group than in the E2 + ConD group (*P* < 0.01) (Figure 3A, left). Locomotor activity during the light phase was significantly lower in the Veh + EqD group than in the Veh + ConD group (*P* < 0.01), whereas activity in the E2 + EqD group did not differ from that in the E2 + ConD group (Figure 3B, left). In addition, the activity in the Veh + EqD group did not differ from that in the E2 + ConD or E2 + EqD group (Figure 3A, left). Locomotor activity did not differ among the four groups during the dark phase (Figure 3A, right).

Daily activity did not differ among the four experimental groups (Figure 3B). The percentage of daily activity during the light phase was higher in the Veh + ConD group than in the E2 + ConD group (*P* < 0.05). The percentage of daily activity during the light phase was lower in the Veh + EqD group than in the Veh + ConD group (*P* < 0.05), whereas it was similar between the E2 + EqD and E2 + ConD groups and between the Veh + EqD and E2 + EqD groups (Figure 3C).

### c-Fos Expression in the SCN

**Figure 4:**
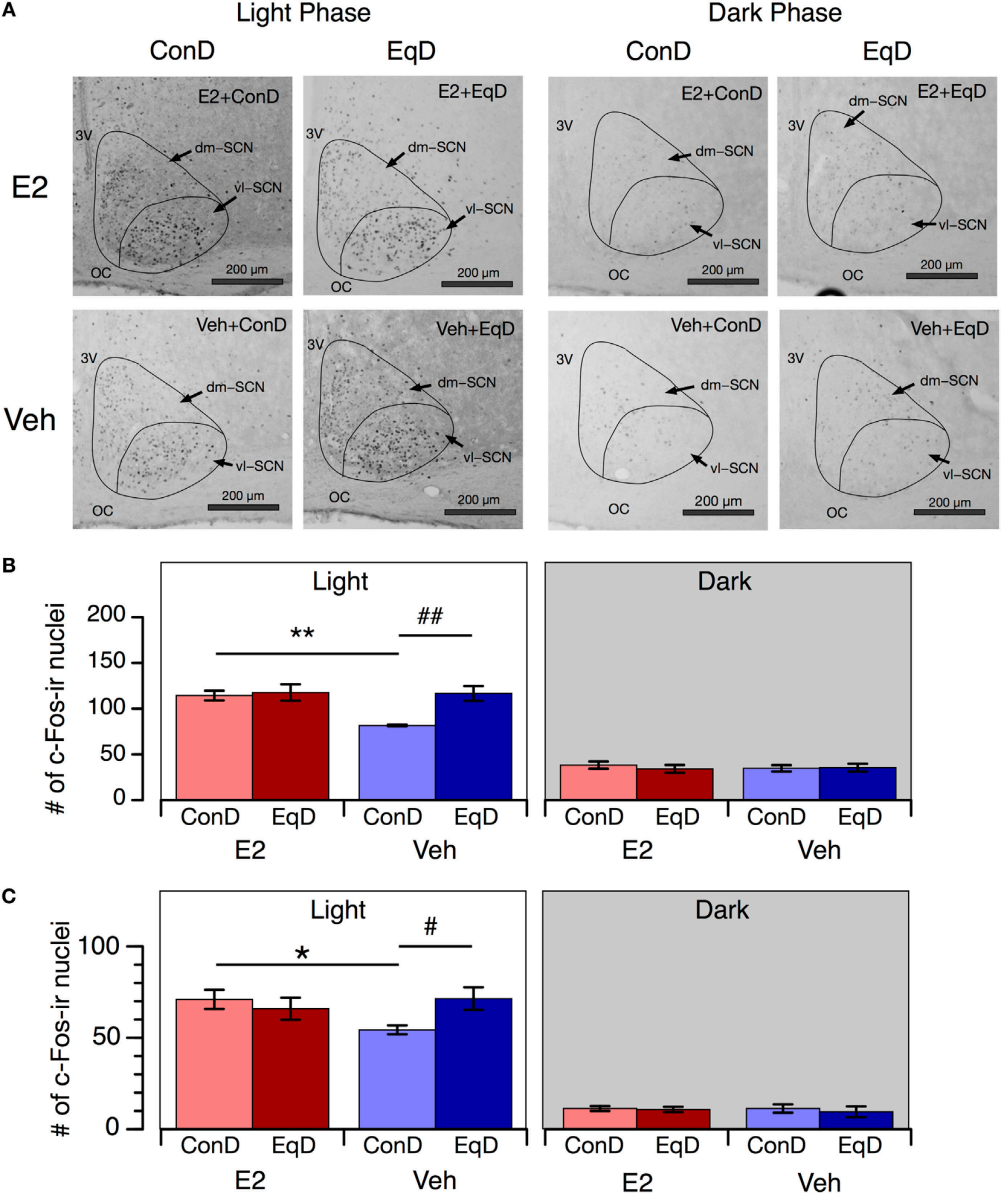
The effect of estradiol (E2) replacement and dietary *S*-equol on c-Fos expression in the suprachiasmatic nucleus (SCN). Rats were ovariectomized and assigned to the E2-replaced or cholesterol [vehicle (Veh)]-treated group. These rats were fed a standard (control) diet (ConD) or an *S*-equol-containing diet (EqD). Samples were obtained on the 13th day after ovariectomy. **(A)** Representative light microscopy images of c-Fos-immunoreactive nuclei in the SCN in estradiol-replaced (E2) and vehicle-treated (Veh) rats fed a control (ConD) or an *S*-equol-containing (EqD) diet during the light [Zeitgeber time (ZT) 2, 0900 h] and dark (ZT14, 2100 h) phases. vl-SCN, ventrolateral SCN; dm-SCN, dorsomedial SCN; OC, optic chiasm; and 3V, third ventricle. Scale bars: 200 µm. **(B)** The number of c-Fos immunoreactive (c-Fos-ir) nuclei in the whole SCN. **(C)** The number of c-Fos-ir nuclei in the ventrolateral SCN. Values are shown as the mean ± SEM (ZT2: *n* = 5 in E2 + ConD and E2 + EqD; *n* = 6 in Veh + ConD; *n* = 7 in Veh + EqD; ZT14: *n* = 5 in each group). * and **, significant differences (*P* < 0.05 and *P* < 0.01, respectively) between the E2-replaced and Veh-treated groups fed the same diet; ^#^ and ^##^, significant differences (*P* < 0.05 and *P* < 0.01, respectively) between the rats fed ConD and EqD with the same treatment.

The number of c-Fos-ir nuclei in the SCN showed a clear circadian rhythm in all groups: more c-Fos-ir nuclei were observed in the SCN during the light phase than during the dark phase (Figures 4A,B). During the light phase, significantly fewer c-Fos-ir nuclei were observed in the whole SCN in the Veh + ConD group than in the E2 + ConD group (*P* < 0.01) (Figure 4B). More c-Fos-ir nuclei were observed in the SCN during the light phase in the Veh + EqD group than in the Veh + ConD group (*P* < 0.01), whereas the number of c-Fos-ir nuclei was similar between the E2 + EqD and E2 + ConD groups and the Veh + EqD and E2 + EqD groups (Figure 4B). During the dark phase, the number of c-Fos-ir nuclei in the SCN did not differ among the four groups.

As in the whole SCN, the numbers of c-Fos-ir nuclei in the ventrolateral SCN also showed a clear circadian rhythm in all groups, and the effects of E2 replacement and dietary *S*-equol on the number of c-Fos-ir nuclei in these regions were similar to those in the whole SCN (Figure 4C).

### ER-α Expression in the MPO and Arc

**Figure 5:**
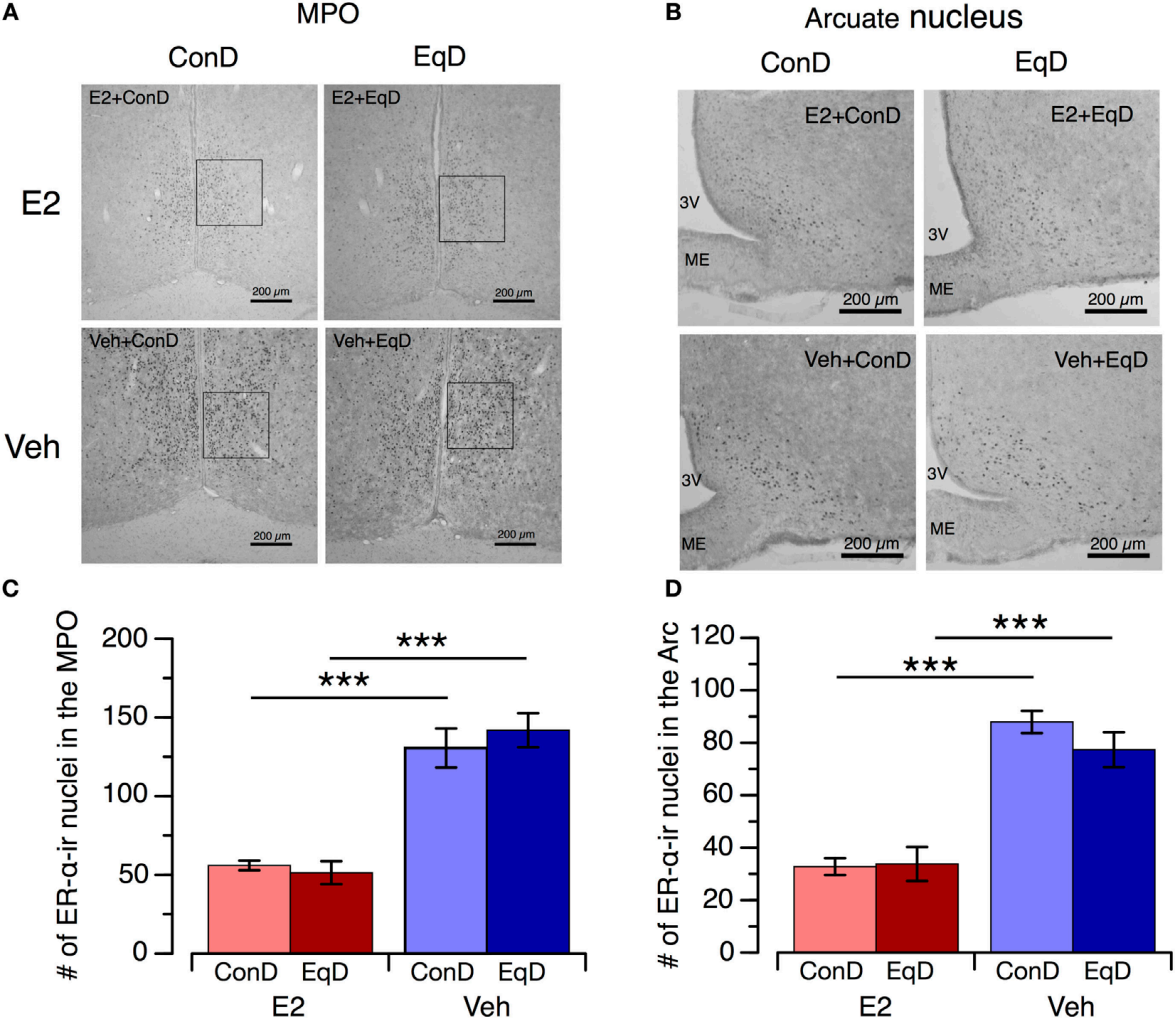
The effect of estradiol (E2) replacement and dietary *S*-equol on estrogen receptor-α (ER-α) expression in the medial preoptic area (MPO) and arcuate nucleus (Arc). Samples were obtained on the 13th day after ovariectomy. Rats were ovariectomized and assigned to the E2-replaced or cholesterol [vehicle (Veh)]-treated group. These rats were fed a standard (control) diet (ConD) or an *S*-equol-containing diet (EqD). Samples were obtained on the 13th day after ovariectomy. **(A,B)** Representative light microscopy images of ER-α-immunoreactive (ir) nuclei in the MPO **(A)** and Arc **(B)** in estradiol-replaced (E2) and vehicle-treated (Veh) rats fed a control (ConD) or an *S*-equol-containing (EqD) diet. 3 V, third ventricle; ME, median eminence; Scale bars: 200 µm. C and D: the number of ER-α-ir cells in the MPO **(C)** and Arc **(D)**. Values are shown as the mean ± SEM (*n* = 10 in E2 + ConD; *n* = 10 in E2 + EqD; *n* = 11 in Veh + ConD; *n* = 12 in Veh + EqD). ***, indicates a significant difference (*P* < 0.001) between the E2-replaced and Veh-treated rats fed the same diet.

The number of ER-α-ir nuclei in the MPO and Arc was greater in the Veh + ConD group than the E2 + ConD group (*P* < 0.001) and in the Veh + EqD group than the E2 + EqD group (*P* < 0.001) (Figure 5). By contrast, the number of ER-α-ir nuclei in the MPO and Arc did not differ between the E2 + ConD and E2 + EqD groups or between the Veh + ConD and Veh + EqD groups (Figure 5).

### Endometrial Epithelial Layer Thickness, Pituitary Gland Weight, and Plasma Glucose Concentration

**Figure 6:**
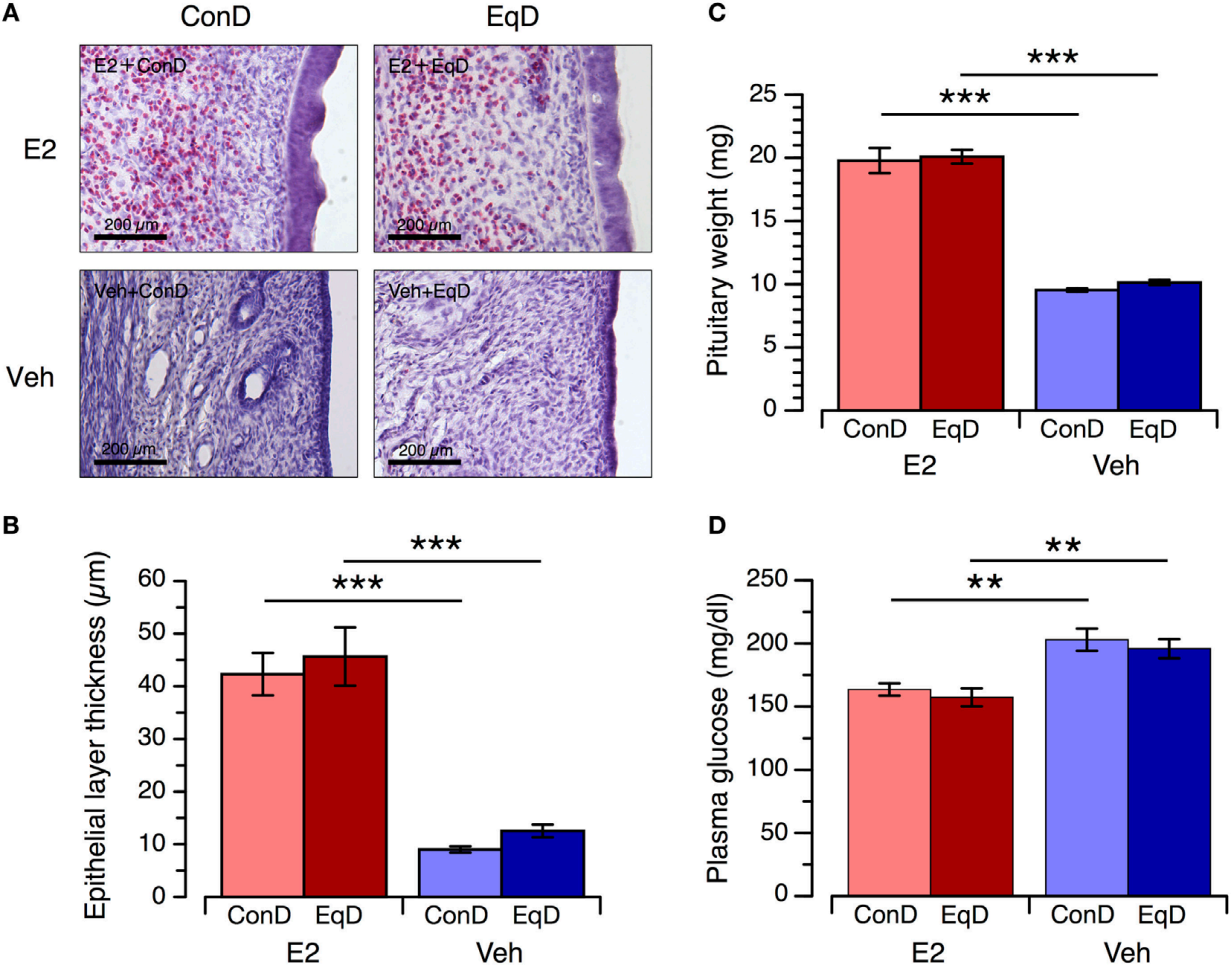
The effect of estradiol (E2) replacement and dietary *S*-equol on the endometrial epithelial layer thickness, pituitary gland weight and plasma glucose concentration. Rats were ovariectomized and assigned to the E2-replaced or cholesterol [vehicle (Veh)]-treated group. These rats were fed a standard (control) diet (ConD) or an *S*-equol-containing diet (EqD). Samples were obtained on the 13th day after ovariectomy. **(A)** Representative light microscopy images of the epithelial layer of the endometrium in the estradiol-replaced (E2) and Veh-treated rats fed a control (ConD) or an *S*-equol-containing (EqD) diet. B, C and D: the endometrial epithelial layer thickness **(B)**, pituitary gland weight **(C)** and plasma glucose concentration **(D)**. Values are shown as the mean ± SEM (*n* = 10 in E2 + ConD; *n* = 10 in E2 + EqD; *n* = 11 in Veh + ConD; *n* = 12 in Veh + EqD). ***, indicates a significant difference (*P* < 0.001) between the E2-replaced and Veh-treated rats fed the same diet.

The thickness of the endometrial epithelial layer and weight of the pituitary gland were significantly greater in the E2 + ConD group than the Veh + ConD group (*P* < 0.001 and *P* < 0.001, respectively) and in the E2 + EqD group than the Veh + EqD group (*P* < 0.001 and *P* < 0.001, respectively) (Figures 6A,B). The thickness of the endometrial epithelial layer and weight of the pituitary gland did not differ between the E2 + ConD and E2 + EqD groups or between the Veh + ConD and Veh + EqD groups (Figure 6C).

The plasma glucose concentration was significantly lower in the E2 + ConD group than the Veh + ConD group and in the E2 + EqD group than the Veh + EqD group. The plasma glucose concentration did not differ between the E2 + ConD and E2 + EqD groups or between the Veh + ConD and Veh + EqD groups (Figure 6D).

## Discussion

The findings of the present study demonstrate that dietary *S*-equol administration exerts its anorectic effect through an estrogen-like action on circadian feeding rhythm regulation and suggest that this effect is unlikely to be mediated by ER-α but by ER-β or through other mechanisms.

Our data confirmed that *S*-equol exerts an estrogen-like anorectic effect. Dietary *S*-equol reduced food intake in Veh-treated rats but not in E2-replaced rats, i.e., the anorectic effects of E2 replacement and dietary *S*-equol were not additive. These findings suggest that the mechanism underlying the anorectic effect of *S*-equol is not independent of the mechanism responsible for estrogen-induced anorexia.

We also found that dietary *S*-equol modified the diurnal feeding pattern in a manner similar to E2 replacement (28, 29). Dietary *S*-equol attenuated food intake and enhanced c-Fos expression in Veh-treated rats but not in E2-replaced rats, specifically during the light phase. These results suggest that dietary *S*-equol has an anorectic effect through estrogen-like action on circadian feeding rhythm regulation, i.e., *S*-equol reduces food intake by modifying feeding behavior during the light phase.

Because taste preference is a critical determining factor in food intake, the reduction in food intake achieved by dietary *S*-equol may be caused by the taste of the EqD (6). It is possible that estrogen deficiency attenuates the palatability of the EqD because the anorectic effect of *S*-equol was observed only in the Veh-treated rats but not in the E2-replaced rats, and estrogen might modify taste preference (6). However, the anorectic effect of dietary *S*-equol in the Veh-treated rats was observed only during the light phase. Thus, it is highly unlikely that the taste of the EqD was the main factor underlying *S*-equol-induced hypophagia.

Another important finding of the present study is that ER-α is unlikely to be involved in the anorectic effect of *S*-equol. ER-α expression in the MPO and Arc was lower in the E2 + ConD group than in the Veh + ConD group, indicating that E2 replacement downregulates ER-α expression in the MPO and Arc in ovariectomized rats. This result is consistent with the findings of other studies (30). However, dietary *S*-equol did not alter the expression of ER-α in the MPO or Arc in either the E2-replaced or Veh-treated animals. It is likely that *S*-equol does not stimulate ER-α in the MPO or Arc, resulting in a lack of ER-α downregulation, although it is unclear whether the ER-α downregulation induced by E2 replacement is mediated by ER-α stimulation (36). In any case, the anorectic effects of E2 and *S*-equol were similar under the experimental conditions of this study, whereas ER-α expression was much greater in the Veh + EqD group than in the E2-replaced groups, suggesting that ER-α is unlikely to be involved in the anorectic effect of *S*-equol.

Furthermore, E2 replacement increased the endometrial epithelial layer thickness and pituitary gland weight, whereas *S*-equol administration did neither (based on the two-way ANOVA results). These results suggest that *S*-equol exerts no hypertrophic actions on the endometrium or the pituitary gland. However, comparing the thickness of the endometrial epithelial layer and weight of the pituitary gland between the Veh + ConD and Veh + EqD groups using an unpaired *t*-test revealed a significant difference. If present, the hypertrophic actions of *S*-equol on the endometrium and pituitary gland at the dose employed in the present study were extremely small (32, 37) compared with the effect of E2, whereas the anorectic action of dietary *S*-equol was similar to that of E2 replacement. These observations also suggest that dietary *S*-equol reduces food intake with minimal stimulation of ER-α and that ER-β or other mechanisms, such as peroxisome proliferator-activated receptors or membrane-bound ERs, may be involved in *S*-equol-induced hypophagia (17, 38). Moreover, because our data suggest that the same mechanism is involved in both E2-induced and *S*-equol-induced hypophagia, we speculate that E2-induced hypophagia is not primarily mediated by ER-α (17, 28).

Our data suggest that modification of the diurnal feeding pattern by both *S*-equol and E2 is achieved by modifying the SCN neuronal activity rhythm. Both E2 and *S*-equol attenuated locomotor activity as well as food intake and enhanced c-Fos expression in the SCN, specifically during the light phase. Thus, *S*-equol and E2 do not act specifically on circadian feeding rhythm regulation but more globally on regulation of the central circadian rhythm (39, 40). It is plausible that estrogen and *S*-equol directly or indirectly modify the circadian rhythm of SCN neuronal activity, resulting in modification of the circadian feeding rhythm and locomotor activity (41, 42). Estrogen and *S*-equol might directly modify the activity of cells in the SCN controlling circadian rhythm, because the most of the pacemaker cells of the SCN are reported to preferentially express ER-β (43). Other possible sites of E2 and *S*-equol action that may lead to enhanced SCN activity are ER-rich brain sites outside the SCN sending afferent signals to the SCN, such as the dorsal and median raphe, preoptic area, corticomedial amygdala, and bed nucleus of the stria terminalis (44–46). However, it is also possible that estrogen and *S*-equol act on targets outside the SCN that modify SCN neuronal activity, feeding, and locomotor activity in parallel (41).

In the present study, an EqD was prepared by adding the *S-*equol-containing soy product SE5-OH to the control diet instead of adding pure *S*-equol to the control diet. The EqD contains 14.0 mg/kg genistein in addition to 137.5 mg/kg *S*-equol, and we did not add the same amount of genistein to the control diet. Thus, the genistein in the EqD may be involved in the estrogen-like hypophagic action of the EqD. However, Yoneda et al. (30) reported that the food intake was similar in rats fed a modified AIN-93M diet with SE5-OH and a diet with pure *S*-equol. Moreover, the dose of genistein in the present study (1.2 mg/kg/day in the E2 + EqD group and 1.1 mg/kg/day in the Veh + EqD group) is likely to be too small to exert anorectic action. A study reported that orally administered genistein at a dose of ~4.8 mg/kg/day did not alter food intake in intact and ovariectomized Wistar Kyoto rats (47). Another study reported that dietary genistein at 150 mg/kg did not lead to hypophagia in mice, whereas dietary genistein at 1,500 mg/kg did lead to hypophagia (48). Thus, in the present study, the contribution of genistein to the estrogen-like hypophagic action of the EqD (138.7 mg/kg) seems to be sufficiently small, although the synergistic effect of genistein with *S*-equol on food intake cannot be completely ruled out.

The mean daily *S*-equol intake over the 7-day measurement period was 11.14 ± 0.29 mg/kg/day in the E2 + EqD group and 10.54 ± 0.26 mg/kg/day in the Veh + EqD group. This dose has been reported to effectively reduce tail skin temperature, an animal model of hot flashes, and food intake in ovariectomized rats (22). Our data show that this dose of dietary *S*-equol has an effect on feeding behavior similar to that of the proestrus plasma E2 level, whereas *S*-equol exerts less antiobesity activity than E2. Furthermore, in the present study, E2 replacement reduced the plasma glucose concentration, but dietary *S*-equol did not. These findings suggest that at the dose used in the present study, the metabolic actions of *S*-equol are weaker than those of E2.

Usui, et al. (27) reported that *S*-equol supplementation at a dose of 10 mg/day was effective to improve HbA1c and low-density lipoprotein cholesterol in obese/overweight Japanese individuals, including men and women, with a mean age of 60 years. They also reported that *S*-equol was more effective in women of equol non-producer. However, they failed to find the antiobesity effect of *S*-equol with that dose, and did not examine the effect of *S*-equol on food intake. The effective dose of *S*-equol that prevents hyperphagia and obesity in postmenopausal women remains unknown. Further study is expected to be done to clarify the effective dose of S-equol that prevents obesity and hyperphagia in postmenopausal women.

In conclusion, *S*-equol exerts an estrogen-like anorectic action by modifying the diurnal feeding pattern. The mechanism of *S*-equol-induced anorexia is unlikely to be mediated by ER-α but rather by ER-β or through other mechanisms, and *S*-equol exerts no hypertrophic actions on the endometrium or the pituitary gland. Our data suggest a possibility that dietary *S*-equol could be an alternative to HRT for the prevention of hyperphagia, obesity, and metabolic syndrome, as well as the relief of perimenopausal symptoms with a lower risk of adverse effects, such as endometrial cancer.

## Ethics Statement

All experiments and animal care procedures were conducted in accordance with the guidelines for animal care and use of Nara Women’s University. All experimental procedures were approved by the Animal Care and Use Committee of Nara Women’s University.

## Author Contributions

YN, KM, AzT, and AkT designed the research; YN, KM, AzT, YH, and AkT conducted the experiment; TU and SU designed the formula for and provided essential information regarding the *S*-equol-containing diet; YN, KM, AzT, HN, KM, TU, SU, and AkT analyzed the data; YN, KT, AzT, and AkT wrote the article; AkT had primary responsibility for the final content. All authors read and approved the final manuscript.

## Conflict of Interest Statement

The authors declare that the research was conducted in the absence of any commercial or financial relationships that could be construed as a potential conflict of interest.

## References

[1] Brown LM Clegg DJ. Central effects of estradiol in the regulation of food intake, body weight, and adiposity. J Steroid Biochem Mol Biol (2010) 122:65–73. 10.1016/j.jsbmb.2009.12.005 20035866 PMC2889220

[2] Jordan VC. Selective estrogen receptor modulation: concept and consequences in cancer. Cancer Cell (2004) 5:207–13. 10.1016/s1535-6108(04)00059-5 15050912

[3] Lobo RA. Metabolic syndrome after menopause and the role of hormones. Maturitas (2008) 60:10–8. 10.1016/j.maturitas.2008.02.008 18407440

[4] Manson JE Colditz GA Stampfer MJ Willett WC Rosner B Monson RR A prospective study of obesity and risk of coronary heart disease in women. N Engl J Med (1990) 322:882–9. 10.1056/NEJM199003293221303 2314422

[5] Poehlman ET Menopause, energy expenditure, and body composition. Acta Obstet Gynecol Scand (2002) 81:603–11. 10.1080/j.1600-0412.2002.810705.x 12190834

[6] Asarian L Geary N. Sex differences in the physiology of eating. Am J Physiol Regul Integr Comp Physiol (2013) 305:R1215–67. 10.1152/ajpregu.00446.2012 23904103 PMC3882560

[7] Mauvais-Jarvis F Clegg DJ Hevener AL The role of estrogens in control of energy balance and glucose homeostasis. Endocr Rev (2013) 34:309–38. 10.1210/er.2012-1055 23460719 PMC3660717

[8] Eckel LA. The ovarian hormone estradiol plays a crucial role in the control of food intake in females. Physiol Behav (2011) 104:517–24. 10.1016/j.physbeh.2011.04.014 21530561 PMC3139826

[9] Foryst-Ludwig A Kintscher U. Metabolic impact of estrogen signalling through ERalpha and ERbeta. J Steroid Biochem Mol Biol (2010) 122:74–81. 10.1016/j.jsbmb.2010.06.012 20599505

[10] Santollo J Eckel LA. Effect of a putative ERalpha antagonist, MPP, on food intake in cycling and ovariectomized rats. Physiol Behav (2009) 97:193–8. 10.1016/j.physbeh.2009.02.021 19254732 PMC2699763

[11] Santollo J Wiley MD Eckel LA. Acute activation of ER alpha decreases food intake, meal size, and body weight in ovariectomized rats. Am J Physiol Regul Integr Comp Physiol (2007) 293:R2194–201. 10.1152/ajpregu.00385.2007 17942491

[12] Liang Y-Q Akishita M Kim S Ako J Hashimoto M Iijima K Estrogen receptor beta is involved in the anorectic action of estrogen. Int J Obes Relat Metab Disord (2002) 26:1103–9. 10.1038/sj.ijo.0802054 12119576

[13] Kristensen K Pedersen SB Vestergaard P Mosekilde L Richelsen B. Hormone replacement therapy affects body composition and leptin differently in obese and non-obese postmenopausal women. J Endocrinol (1999) 163:55–62. 10.1677/joe.0.1630055 10495407

[14] Sumino H Ichikawa S Yoshida A Murakami M Kanda T Mizunuma H Effects of hormone replacement therapy on weight, abdominal fat distribution, and lipid levels in Japanese postmenopausal women. Int J Obes Relat Metab Disord (2003) 27:1044–51. 10.1038/sj.ijo.0802371 12917709

[15] Dahlman-Wright K Cavailles V Fuqua SA Jordan VC Katzenellenbogen JA Korach KS International Union of Pharmacology. LXIV. Estrogen receptors. Pharmacol Rev (2006) 58:773–81. 10.1124/pr.58.4.8 17132854

[16] Chen M-N Lin C-C Liu C-F. Efficacy of phytoestrogens for menopausal symptoms: a meta-analysis and systematic review. Climacteric (2015) 18:260–9. 10.3109/13697137.2014.966241 25263312 PMC4389700

[17] Jungbauer A Medjakovic S. Phytoestrogens and the metabolic syndrome. J Steroid Biochem Mol Biol (2014) 139:277–89. 10.1016/j.jsbmb.2012.12.009 23318879

[18] Ørgaard A Jensen L The effects of soy isoflavones on obesity. Exp Biol Med (Maywood) (2008) 233:1066–80. 10.3181/0712-MR-347 18535167

[19] Dewi FN Wood CE Lampe JW Hullar MAJ Franke AA Golden DL Endogenous and exogenous equol are antiestrogenic in reproductive tissues of apolipoprotein e-null mice. J Nutr (2012) 142:1829–35. 10.3945/jn.112.161711 22933749 PMC3442795

[20] Jackson RL Greiwe JS Schwen RJ. Emerging evidence of the health benefits of S-equol, an estrogen receptor β agonist. Nutr Rev (2011) 69:432–48. 10.1111/j.1753-4887.2011.00400.x 21790611

[21] Setchell KDR Clerici C. Equol: history, chemistry, and formation. J Nutr (2010) 140:1355S–62S. 10.3945/jn.109.119776 20519412 PMC2884333

[22] Yoneda T Ueno T Uchiyama S. S-equol and the fermented soy product SE5-OH containing S-equol similarly decrease ovariectomy-induced increase in rat tail skin temperature in an animal model of hot flushes. Menopause (2011) 18:814–20. 10.1097/gme.0b013e318208fb0d 21451423

[23] Ishimi Y. Dietary equol and bone metabolism in postmenopausal Japanese women and osteoporotic mice. J Nutr (2010) 140:1373S–6S. 10.3945/jn.110.124842 20484547

[24] Legette LL Martin BR Shahnazari M Lee W-H Helferich WG Qian J Supplemental dietary racemic equol has modest benefits to bone but has mild uterotropic activity in ovariectomized rats. J Nutr (2009) 139:1908–13. 10.3945/jn.109.108225 19710157 PMC2744611

[25] Blake C Fabick KM Setchell KD Lund TD Lephart ED. Neuromodulation by soy diets or equol: anti-depressive & anti-obesity-like influences, age- & hormone-dependent effects. BMC Neurosci (2011) 12:28. 10.1186/1471-2202-12-28 21410981 PMC3068123

[26] Rachoń D Vortherms T Seidlová-Wuttke D Wuttke W. Effects of dietary equol on body weight gain, intra-abdominal fat accumulation, plasma lipids, and glucose tolerance in ovariectomized Sprague-Dawley rats. Menopause (2007) 14:925–32. 10.1097/GME.0b013e31802d979b 17414092

[27] Usui T Tochiya M Sasaki Y Muranaka K Yamakage H Himeno A Effects of natural S-equol supplements on overweight or obesity and metabolic syndrome in the Japanese, based on sex and equol status. Clin Endocrinol (Oxf) (2013) 78:365–72. 10.1111/j.1365-2265.2012.04400.x 22469418

[28] Takamata A Torii K Miyake K Morimoto K. Chronic oestrogen replacement in ovariectomised rats attenuates food intake and augments c-Fos expression in the suprachiasmatic nucleus specifically during the light phase. Br J Nutr (2011) 106:1283–9. 10.1017/S0007114511001607 21736812

[29] Varma M Chai JK Meguid MM Laviano A Gleason JR Yang ZJ Effect of estradiol and progesterone on daily rhythm in food intake and feeding patterns in Fischer rats. Physiol Behav (1999) 68:99–107. 10.1016/s0031-9384(99)00152-3 10627068

[30] Yamada S Noguchi D Ito H Yamanouchi K. Sex and regional differences in decrease of estrogen receptor alpha-immunoreactive cells by estrogen in rat hypothalamus and midbrain. Neurosci Lett (2009) 463:135–9. 10.1016/j.neulet.2009.07.074 19646508

[31] Scully KM Gleiberman AS Lindzey J Lubahn DB Korach KS Rosenfeld MG Role of estrogen receptor-alpha in the anterior pituitary gland. Mol Endocrinol (1997) 11:674–81. 10.1210/mend.11.6.0019 9171231

[32] Rachoń D Vortherms T Seidlová-Wuttke D Menche A Wuttke W Uterotropic effects of dietary equol administration in ovariectomized Sprague-Dawley rats. Climacteric (2007) 10:416–26. 10.1080/13697130701624757 17852145

[33] Yee S Burdock GA Kurata Y Enomoto Y Narumi K Hamada S Acute and subchronic toxicity and genotoxicity of SE5-OH, an equol-rich product produced by *Lactococcus garvieae*. Food Chem Toxicol (2008) 46:2713–20. 10.1016/j.fct.2008.04.026 18554770

[34] Paxinos G Watson C The Rat Brain in Stereotaxic Coordinates. 4th ed San Diego: Academic Press (2004).

[35] Butcher RL Collins WE Fugo NW Plasma concentration of LH, FSH, prolactin, progesterone and estradiol-17beta throughout the 4-day estrous cycle of the rat. Endocrinology (1974) 94:1704–8. 10.1210/endo-94-6-1704 4857496

[36] Schreihofer DA Stoler MH Shupnik MA. Differential expression and regulation of estrogen receptors (ERs) in rat pituitary and cell lines: estrogen decreases ERalpha protein and estrogen responsiveness. Endocrinology (2000) 141:2174–84. 10.1210/endo.141.6.7505 10830306

[37] Rachoń D Vortherms T Seidlová-Wuttke D Wuttke W. Effects of dietary equol on the pituitary of the ovariectomized rats. Horm Metab Res (2007) 39:256–61. 10.1055/s-2007-973074 17447162

[38] Santollo J Marshall A Daniels D. Activation of membrane-associated estrogen receptors decreases food and water intake in ovariectomized rats. Endocrinology (2013) 154:320–9. 10.1210/en.2012-1858 23183173 PMC3529383

[39] Bailey M Silver R. Sex differences in circadian timing systems: implications for disease. Front Neuroendocrinol (2014) 35:111–39. 10.1016/j.yfrne.2013.11.003 24287074 PMC4041593

[40] Kuljis DA Loh DH Truong D Vosko AM Ong ML McClusky R Gonadal- and sex-chromosome-dependent sex differences in the circadian system. Endocrinology (2013) 154:1501–12. 10.1210/en.2012-1921 23439698 PMC3602630

[41] Bechtold DA Loudon ASI. Hypothalamic clocks and rhythms in feeding behaviour. Trends Neurosci (2013) 36:74–82. 10.1016/j.tins.2012.12.007 23333345

[42] Krajnak K Kashon ML Rosewell KL Wise PM. Sex differences in the daily rhythm of vasoactive intestinal polypeptide but not arginine vasopressin messenger ribonucleic acid in the suprachiasmatic nuclei. Endocrinology (1998) 139:4189–96. 10.1210/endo.139.10.6259 9751499

[43] Vida B Hrabovszky E Kalamatianos T Coen CW Liposits Z Kalló I. Oestrogen receptor alpha and beta immunoreactive cells in the suprachiasmatic nucleus of mice: distribution, sex differences and regulation by gonadal hormones. J Neuroendocrinol (2008) 20:1270–7. 10.1111/j.1365-2826.2008.01787.x 18752649

[44] Karatsoreos IN Silver R. Minireview: the neuroendocrinology of the suprachiasmatic nucleus as a conductor of body time in mammals. Endocrinology (2007) 148:5640–7. 10.1210/en.2007-1083 17901227 PMC3423957

[45] Abizaid A Mezei G Thanarajasingam G Horvath TL. Estrogen enhances light-induced activation of dorsal raphe serotonergic neurons. Eur J Neurosci (2005) 21:1536–46. 10.1111/j.1460-9568.2005.03964.x 15845081

[46] Abizaid A Mezei G Horvath TL. Estradiol enhances light-induced expression of transcription factors in the SCN. Brain Res (2004) 1010:35–44. 10.1016/j.brainres.2004.01.089 15126115

[47] Bitto A Altavilla D Bonaiuto A Polito F Minutoli L Di Stefano V Effects of aglycone genistein in a rat experimental model of postmenopausal metabolic syndrome. J Endocrinol (2009) 200:367–76. 10.1677/JOE-08-0206 19066292

[48] Kim H-K Nelson-Dooley C Della-Fera MA Yang J-Y Zhang W Duan J Genistein decreases food intake, body weight, and fat pad weight and causes adipose tissue apoptosis in ovariectomized female mice. J Nutr (2006) 136:409–14. 10.1038/oby.2005.189 16424120

