# Supplementary material for: S-equol Exerts Estradiol-Like Anorectic Action with Minimal Stimulation of Estrogen Receptor-α in Ovariectomized Rats
Source: Front Endocrinol (Lausanne). 2017 Oct 19;8:281. doi: 10.3389/fendo.2017.00281 (PMC5653693; doi:10.3389/fendo.2017.00281)
Supplement: Supplementary file 1 [file table_1.docx]

Supplementary Material

*S*-equol Exerts Estradiol-like Anorectic Action with Minimal Stimulation of Estrogen Receptor-α in Ovariectomized Rats

**Yuri Nishimura^1^, Kaori Mabuchi^1^, Azusa Takano^1^, Yayoi Hara^1^, Hiroko Negishi^1^, Keiko Morimoto^1^, Tomomi Ueno^2^, Shigeto Uchiyama^2^, Akira Takamata^1*^**

*** Correspondence:** Akira Takamata: takamata@cc.nara-wu.ac.jp

# Supplementary Tables 1

Contents of isoflavones and metabolites in *SE5-OH* (mg/g) and EqD (mg/kg)

|  | SE5-OH | EqD |
| --- | --- | --- |
|  | (mg/g) | (mg/kg) |
| *S*-equol | 6.03 | 138.69 |
| Daidzin | 0.12 | 2.76 |
| Malonyldaidzin | 0.00 | 0.00 |
| Acetyldaidzin | 0.09 | 2.07 |
| Daidzein | 0.00 | 0.00 |
| Dihydrodaidzein | 0.03 | 0.69 |
| Genistin | 0.12 | 2.76 |
| Malonylgenistin | 0.00 | 0.00 |
| Acetylgenistin | 0.00 | 0.00 |
| Genistein | 0.61 | 14.03 |
| Dihydrogenistein | 1.35 | 31.05 |
| Glycitin | 2.18 | 50.14 |
| Malonylglycitin | 0.00 | 0.00 |
| Acetylglycitin | 0.08 | 1.84 |
| Glycitein | 1.04 | 23.92 |
| Dihydroglycitein | 0.00 | 0.00 |
